# 在线固相萃取净化-超高效液相色谱-串联质谱法测定海洋沉积物中22种抗生素

**DOI:** 10.3724/SP.J.1123.2024.08001

**Published:** 2025-08-08

**Authors:** Lanxiang WANG, Junhui CHEN, Cancan SHENG, Shengqing FAN, Xiuping HE, Xianguo LI

**Affiliations:** 1.中国海洋大学，海洋化学理论与工程技术教育部重点实验室，山东 青岛 266100; 1. Key Laboratory of Marine Chemistry Theory and Technology，Ministry of Education，Ocean University of China，Qingdao 266100，China; 2.自然资源部第一海洋研究所，青岛市现代分析与近海生态环境安全保障重点实验室，山东 青岛 266061; 2. Qingdao Key Laboratory of Analytical Technology Development and Offshore Eco-Environment Conservation，First Institute of Oceanography，Ministry of Natural Resources，Qingdao 266061，China; 3.青岛海洋科技中心，海洋生态与环境科学功能实验室，山东 青岛 266071; 3. Laboratory for Marine Ecology and Environmental Science，Qingdao Marine Science and Technology Center，Qingdao 266071，China

**Keywords:** 在线固相萃取, 超高效液相色谱-串联质谱, 沉积物, 抗生素, 四十里湾, online solid-phase extraction（SPE）, ultra-high performance liquid chromatography-tandem mass spectrometry（UHPLC-MS/MS）, sediments, antibiotics, Sishili Bay

## Abstract

本研究将在线固相萃取（SPE）技术与超高效液相色谱-串联质谱（UHPLC-MS/MS）技术结合，建立了一种检测海洋沉积物中22种常见抗生素的新方法，并将该方法用于近海海湾沉积物中抗生素的分析检测。通过对样品提取条件及在线SPE条件的系统优化，获得了最佳实验条件。沉积物样品经乙腈-EDTA/McIlvaine缓冲溶液（1∶1，v/v）提取后，用超纯水稀释，采用以大孔苯乙烯/二乙烯基苯为填料的PLRP-S在线SPE柱净化富集后，通过Poroshell EC-C18色谱柱（50 mm×2.1 mm，1.9 µm）分离；在电喷雾电离、正离子模式下，以多反应监测（MRM）模式进行检测，整个分析流程可在14 min内完成。结果表明，22种抗生素在各自的质量浓度范围内具有良好的线性关系，相关系数（*R*^2^）均≥0.990 0，检出限（LOD，*S/N*=3）为0.001~0.08 ng/g，定量限（LOQ，*S/N*=10）为0.004~0.4 ng/g。在低、中、高3个加标水平下，22种抗生素的加标回收率为45.1%~145.6%，相对标准偏差（RSD）<14%。采用该方法对山东近海四十里湾冬季和夏季沉积物样品中的抗生素进行检测，结果显示，在9个夏季沉积物样品中共检出5类19种抗生素，含量为0.01~34.64 ng/g，其中土霉素的检出水平最高；在10个冬季沉积物样品中共检出5类20种抗生素，含量为0.004~19.11 ng/g，其中氧氟沙星的检出水平最高。与常用的离线SPE方法相比，该方法大大简化了样品的净化处理过程，为海洋沉积物中常见抗生素的日常检测提供了一种简便且有效的方法。

## Abstract (en)

This study developed a novel method for the detection of 22 antibiotics in marine sediments by integrating online solid-phase extraction（SPE） with ultra-high performance liquid chromatography-tandem mass spectrometry（UHPLC-MS/MS）. The method was subsequently applied to the analytical determination of antibiotics in offshore bay sediments. Optimal experimental conditions were established through systematic optimization of sample extraction and online SPE parameters. Sediment samples were extracted using an acetonitrile-EDTA/McIlvaine buffer solution（1∶1， v/v）， diluted with ultrapure water， and then purified and enriched using a large-pore styrene/divinylbenzene-packed PLRP-S online SPE column. The processed samples were separated on a Poroshell EC-C18 column（50 mm×2.1 mm， 1.9 µm） and detected in electrospray ionization（ESI） positive ion mode with multiple reaction monitoring（MRM） acquisition. The entire analytical process was completed within 14 min. The results showed that the 22 antibiotics showed good linearity in their respective mass concentration ranges with the correlation coefficients（*R*^2^）≥0.990 0， the limits of detection（LODs， *S/N*=3） were 0.001‒0.08 ng/g， and the limits of quantification（LOQs， *S/N*=10） were 0.004‒0.4 ng/g. The spiked recoveries of the 22 antibiotics under three spiked levels（low， medium and high） were 45.1%‒145.6% with the relative standard deviations（RSDs）<14%. The method was used to detect antibiotics in winter and summer sediment samples from Shandong Sishili Bay offshore. The results showed that a total of 19 antibiotics from five classes were detected in nine summer sediment samples， with the contents ranging from 0.01 to 34.64 ng/g； and oxytetracycline exhibited the highest detection level. A total of 20 antibiotics from five classes were detected in 10 winter sediment samples， with the contents ranging from 0.004-19.11 ng/g； and ofloxacin showed the highest detection level. Compared with the commonly used offline SPE method， this method greatly simplifies the sample purification process and provides a simple and effective method for the routine detection of common antibiotics in marine sediments.

抗生素主要是天然生成或人工合成的具有抗病原体活性的有机化合物^［1］^。这类物质的抗菌性能优良且价格低廉，不仅被广泛用于人类、牲畜以及水产养殖疾病的治疗，还常被当作饲料添加剂来促进动物生长^［2］^。然而，抗生素在生物体内无法被完全吸收，大约有10%~90%会以原形或代谢产物的形式排出体外，进入环境^［3］^，从而引发抗生素污染问题。近些年来，在海洋、河流、湖泊、土壤、沉积物等各类不同环境介质中，频繁检测出抗生素^［4］^。其中，最常见的抗生素类型主要包括四环素类、磺胺类、喹诺酮类、大环内酯类以及氯霉素类等^［5］^。海洋是抗生素等众多污染物的重要汇聚地^［6］^，尤其是近海环境，由于农业径流、污水处理厂排放等陆源输入，以及海水水产养殖活动等因素，大量抗生素残留在海洋环境中^［3］^。沉积物是海洋环境中抗生素等污染物的储存库^［7］^，Li等^［3］^在东海的杭州湾、香山湾和台州湾的沉积物中检出25种抗生素，总检出含量为2.2~99.9 ng/g，其中红霉素是含量最高的抗生素。Wu等^［8］^在南海北部湾沉积物中检出17种抗生素，总检出含量为1.33~8.55 ng/g，且以诺氟沙星、依诺沙星和恩诺沙星为主。Han等^［9］^在渤海莱州湾夏季沉积物中检出11种抗生素，平均含量为0.03~23.06 ng/g；在渤海莱州湾冬季沉积物中检出10种抗生素，平均含量为2.58~54.04 ng/g，均以恩诺沙星为主。海洋环境中的抗生素会通过迁移、吸附与沉降等过程进入海洋沉积物，并在其中发生累积与释放现象。这些抗生素可能会对贝类、甲壳类等底栖生物产生毒性作用，抑制微生物生长，还会影响底栖微生物的群落结构等^［10］^。基于上述情况，加强对海洋沉积物中常见抗生素的监测工作，阐明近海沉积物中各类抗生素的污染现状，已然势在必行。正因如此，开发一种适用于海洋沉积物中常见抗生素检测的简便方法，就显得尤为必要。

当前，用于抗生素检测的方法主要包括毛细管电泳法^［11］^、酶联免疫法^［12］^、电化学分析法^［13］^、高效液相色谱法^［14］^以及液相色谱-串联质谱法（LC-MS/MS）^［15］^等。在这些方法中，LC-MS/MS法具有检出限（LOD）低、灵敏度高、分析范围广、自动化程度高等优点，是目前应用较为广泛的抗生素检测方法，同时也是海洋环境中抗生素检测领域最为先进的方法。对于沉积物样品中的抗生素，需进行提取和净化处理。常用的提取方法包括超声辅助提取法（UAE）^［16］^、快速溶剂萃取法（ASE）^［17］^、微波萃取法（MAE）^［18］^等。其中，ASE和MAE所需试剂量较少，萃取效率较高，但对时间和温度条件要求严格，若处理时间过长或温度过高，均可能导致目标抗生素降解^［19］^。UAE具有操作简便、试剂用量少、萃取效率高的特点，是目前应用最广泛的提取方法。为了最大限度地减少基质干扰，在提取操作之后，需对提取物进行净化处理。固相萃取（SPE）是常用的净化富集手段^［15］^，该方法可分为离线SPE和在线SPE两种类型。其中，在线SPE技术借助LC的多流路及阀切换技术，可实现SPE过程的全自动化操作^［20］^。与传统的离线SPE方法相比，在线SPE技术展现出更高的自动化程度和更优的经济性，同时还能有效缩短样品制备时间，减少目标化合物的过程损失，并降低溶剂消耗量^［21］^。目前，在线SPE技术已成功应用于海洋生物毒素^［22］^、农药残留^［23］^以及全氟化合物^［24］^等有机分子的在线富集与净化处理。然而，关于将在线SPE技术应用于海洋沉积物中不同种类抗生素的快速净化处理方面的研究，目前尚未见相关报道。

本研究将在线SPE技术与UHPLC-MS/MS技术结合，采用UAE进行快速提取，建立了一种海洋沉积物中5类（喹诺酮类、四环素类、大环内酯类、氯霉素类和磺胺类）22种抗生素的自动化净化检测方法。通过对样品提取条件和在线SPE条件的优化，获得最佳实验条件。将该方法应用于我国山东近海四十里湾沉积物中22种常见抗生素的检测与调查工作，相关研究结果可为深入剖析我国近海沉积物中抗生素的污染特征，以及评估其潜在生态风险提供重要的技术支持和数据参考。

## 1 实验部分

### 1.1 仪器、试剂与材料

1290 Infinity Ⅱ超高效液相色谱仪（配有二元泵、四元泵、柱温箱和自动进样器）和6470型三重四极杆质谱仪（配有喷射流电喷雾电离（AJS-ESI）源，美国Agilent公司）；UNIQUE-R20实验室纯水仪（厦门锐思捷科学仪器有限公司）；Alpha-4LDplus冷冻干燥机（德国CHRIST公司）；FA1104电子天平（上海精天电子仪器厂）；MIX-23P迷你混合仪（杭州迷欧仪器有限公司）；SK3300H型超声波清洗器（上海科导超声仪器有限公司）；HC-3018型离心机（安徽中科中佳科学仪器公司）；DDC2-1箱式采泥器（青岛蓝科海洋仪器设备有限公司）。

22种抗生素标准品：氟罗沙星（FLE，纯度≥98%）购自天津普西唐生物科技有限公司，罗红霉素（ROX，纯度98%）、环丙沙星（CIP，纯度98%）购自上海安耐吉化学有限公司，克拉霉素（CTM，纯度98%）、诺氟沙星（NOR，纯度99%）、甲氧苄啶（TMP，纯度99%）、洛美沙星（LMX，纯度98%）、恩诺沙星（ENR，纯度98%）、磺胺噻唑（STZ，纯度≥98%）购自上海源叶生物科技有限公司，氟苯尼考（FF，纯度98%）、四环素（TET，纯度≥98%）、土霉素（OTC，纯度≥98%）、红霉素（ERY，纯度98%）、氧氟沙星（OFL，纯度98%）、二氟沙星（DFH，纯度98%）、磺胺嘧啶（SDZ，纯度98%）、磺胺二甲基嘧啶（SMZ，纯度99%）购自上海Macklin生化科技有限公司，金霉素（CTC，纯度≥95%）、磺胺甲基嘧啶（SMR，纯度≥98%）、磺胺甲基异恶唑（SMX，纯度≥98%）购自北京Solarbio科技有限公司，培氟沙星（PEF，纯度99%）购自上海阿拉丁生化科技股份有限公司，脱水红霉素（ERY-H_2_O，纯度≥98%）购自天津阿尔塔科技有限公司。其中，FLE、CIP、NOR、LMX、ENR、OFL、DFH和PEF属于喹诺酮类抗生素，ROX、CTM、ERY、ERY-H_2_O属于大环内酯类抗生素，FF属于氯霉素类抗生素，TET、OTC和CTC属于四环素类抗生素，TMP、STZ、SDZ、SMZ、SMR和SMX属于磺胺类抗生素。

同位素内标：CIP-D8、DFH-D3、FLE-D3、LMX-D5、NOR-D5、OFL-D3、ENR-D5和PEF-D5，质量浓度均为100 mg/L（溶剂均为甲醇），购自天津阿尔塔科技有限公司。

乙腈、甲醇和甲酸（色谱纯）购自上海Macklin生化科技有限公司；无水磷酸二氢钠、磷酸氢二钠、乙二胺四乙酸二钠（Na_2_EDTA）、一水合柠檬酸（分析纯）购自国药集团化学试剂有限公司；磷酸（色谱纯）购自天津市科密欧化学试剂有限公司；乙酸铵（质谱纯）购自瑞士Fluka公司；0.22 μm有机滤膜购自津腾实验设备有限公司；实验用水均为实验室自制的超纯水（18.25 MΩ⋅cm）。

### 1.2 溶液的配制

内标混合溶液的配制：分别量取适量的8种同位素内标溶液（100 mg/L），用甲醇逐级稀释至50 µg/L，于4 ℃下避光保存。

混合标准溶液的配制：分别准确称取22种抗生素标准品，用甲醇溶解，配制成质量浓度为100 mg/L的单标储备液；将22种单标储备液混合，用甲醇进行稀释，得到22种抗生素的混合标准储备液（质量浓度为50 µg/L）；用甲醇逐级稀释混合标准储备液，得到系列质量浓度的混合标准溶液，于4 ℃下避光保存。其中，3种四环素类抗生素（CTC、OTC和TET）的质量浓度是其余抗生素质量浓度的10倍。

基质匹配混合标准溶液的配制：用空白沉积物样品的基质提取液配制系列质量浓度（0.5、1、2、4、5、7.5、10、25 ng/L）的基质匹配混合标准溶液，其中3种四环素类抗生素（CTC、OTC和TET）的质量浓度为其余抗生素的10倍。向基质匹配混合标准溶液中加入适量的内标混合溶液，内标的最终质量浓度均为5 ng/L。

磷酸盐缓冲溶液的配制：分别准确称取和量取2.39 g无水磷酸二氢钠和135 µL磷酸，用超纯水溶解定容至100 mL，即得磷酸盐缓冲溶液（pH 3）。

EDTA/McIlvaine缓冲溶液的配制：分别准确称取3.72 g Na_2_EDTA、1.29 g柠檬酸和2.75 g磷酸氢二钠，用超纯水溶解定容至100 mL，即得EDTA/McIlvaine缓冲溶液（pH 4）。

柠檬酸缓冲溶液的配制：分别准确称取0.89 g柠檬酸和1.69 g柠檬酸钠，用超纯水溶解定容至100 mL，即得柠檬酸缓冲溶液（pH 5）^［18］^。

### 1.3 样品的采集与提取

#### 1.3.1 样品采集

分别于2023年7月（夏季）和12月（冬季）在四十里湾采集沉积物样品，采样详细站位如图1所示。其中，夏季沉积物样品采集自S2~S10站点，冬季沉积物采集自S1~S10站点。所有沉积物样品均使用箱式采泥器进行采集，采集后将其置于聚乙烯袋中，并于‒20 ℃环境下冷冻避光保存。样品运回实验室后，采用真空冷冻干燥机进行冷冻干燥处理，冻干后对样品进行研磨并过80目筛，于‒20 ℃条件下保存。

**图1:**
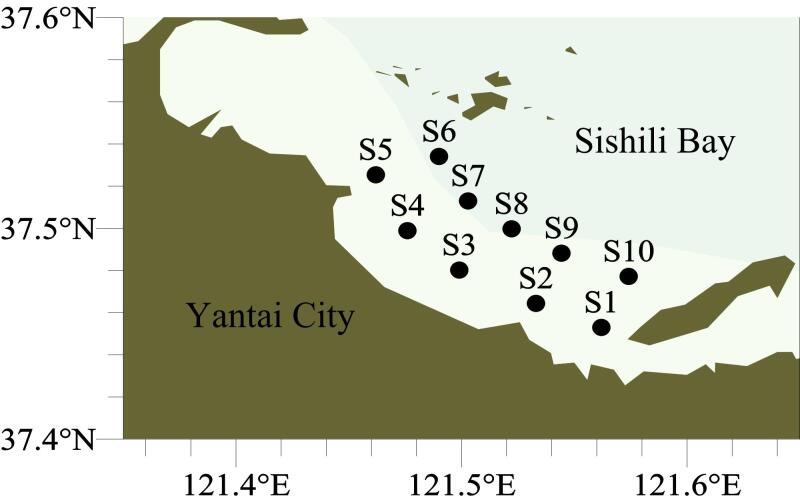
四十里湾采样站位图

#### 1.3.2 样品提取

称取1.00 g上述冻干研磨后的沉积物样品并置于50 mL离心管中，加入100 µL内标混合溶液（50 µg/L），再加入4 mL提取溶剂（乙腈-EDTA/McIlvaine缓冲溶液（1∶1，v/v）），涡旋混匀后用超声辅助提取20 min，在10 000 r/min下离心10 min，并分离出上清液；之后向离心管沉淀中继续加入2 mL乙腈-EDTA/McIlvaine缓冲溶液（1∶1，v/v），按照上述步骤重复提取两次；合并所有的上清液，经超声混匀后过0.22 µm有机滤膜；取滤液100 µL，用超纯水稀释至1 mL，装入UHPLC样品瓶，于‒20 ℃下保存待测。

### 1.4 在线SPE-UHPLC-MS/MS条件

#### 1.4.1 在线SPE条件

采用以大孔苯乙烯/二乙烯基苯为填料的PLRP-S柱（12.5 mm×2.1 mm，15 µm）作为在线SPE柱，进样体积为200 µL。在线SPE操作中两步骤（上样、洗脱与分析）对应的六通阀切换示意图如图2所示。当六通阀的1位与6位相连通时，由四元泵加载流动相，将样品输送至在线SPE柱上，进行目标化合物的在线富集与净化；当六通阀切换至1位与2位相连通时，由二元泵输送的流动相将富集在SPE柱上的目标化合物反冲至分析柱进行分离，随后进入质谱进行检测。

**图2:**
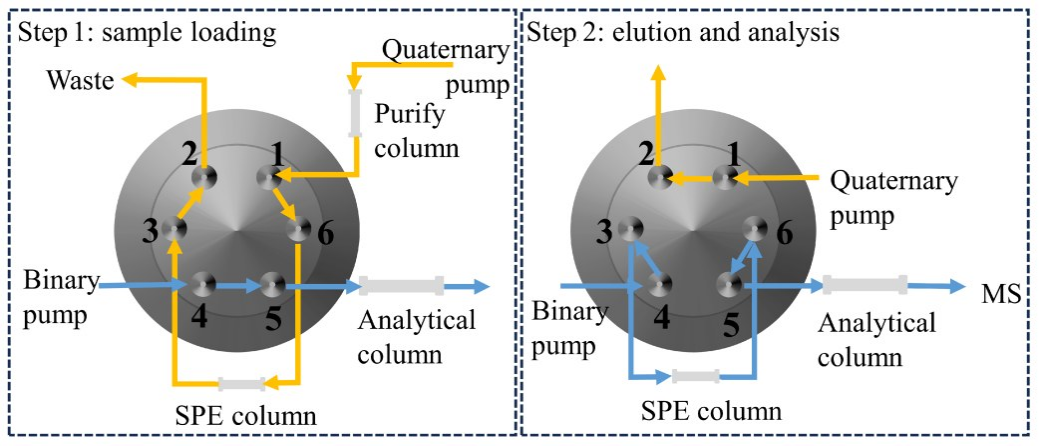
在线SPE六通阀切换示意图

#### 1.4.2 UHPLC-MS/MS条件

分析柱：Poroshell 120 EC-C18柱（50 mm×2.1 mm，1.9 µm，美国Agilent公司）；柱温：25 ℃。四元泵流动相A_1_：0.1%甲酸水溶液（含2.5 mmol/L乙酸铵溶液），流动相B_1_：甲醇，流速1 mL/min；二元泵流动相A_2_：0.1%甲酸水溶液（含2.5 mmol/L乙酸铵溶液），流动相B_2_：乙腈，流速0.3 mL/min。梯度洗脱程序如表1所示。

**表1:** 梯度洗脱程序

Time/min	Quaternary pump			Six-way valve switching	Binary pump			
	*φ*（A_1_）/%	*φ*（B_1_）/%	Flow rate/ （mL/min）		Time/min	*φ*（A_2_）/%	*φ*（B_2_）/%	Flow rate/ （mL/min）
0	98	2	1	1-6	0	90	10	0.3
2	98	2	1	1-2	2	90	10	0.3
3	20	80	1	1-2	5	77	23	0.3
6	20	80	1	1-2	8	40	60	0.3
7	98	2	1	1-2	9	0	100	0.3
9	98	2	1	1-6	11	0	100	0.3
12	98	2	1	1-6	12	90	10	0.3

A_1_： 0.1% formic acid aqueous solution（containing 2.5 mmol/L ammonium acetate solution）； B_1_： methanol； A_2_： 0.1% formic acid aqueous solution（containing 2.5 mmol/L ammonium acetate solution）； B_2_： acetonitrile.

离子源：电喷雾电离（ESI）源，正离子模式；检测模式：多反应监测（MRM）；喷雾气压：241 kPa（N_2_）；毛细管电压：4 000 V；鞘气（N_2_）温度：340 ℃，干燥气（N_2_）温度：300 ℃；鞘气流速：11 L/min，干燥气流速：7 L/min。22种目标抗生素及8种同位素内标的保留时间和质谱参数如表2所示。

**表2:** 22种目标抗生素及8种同位素内标的保留时间及质谱参数

Compound	Molecular formula	*t*_R_/min	Precursor ion（*m/z*）	Product ions（*m/z*）	Fragmentor/V	CEs/eV	Internal standard
Sulfadiazine（SDZ）	C_10_H_10_N_4_O_2_S	3.467	250.9［M+H］^+^	155.9， 92.1^*^	100	13， 33	/
Sulfathiazole（STZ）	C_9_H_9_N_3_O_2_S_2_	3.661	256.0［M+H］^+^	92.0， 156.0^*^	105	13， 29	/
Sulfamerazine（SMR）	C_11_H_12_N_4_O_2_S	3.796	265.0［M+H］^+^	156.0， 92.0^*^	115	17， 33	/
Trimethoprim（TMP）	C_14_H_18_N_4_O_3_	3.872	291.3［M+H］^+^	123.1， 230.1^*^	145	26， 17	/
Oxytetracycline（OTC）	C_22_H_24_N_2_O_9_	4.072	461.2［M+H］^+^	443.0， 426.1^*^	115	11， 21	/
Norfloxacin（NOR）	C_16_H_18_FN_3_O_3_	4.123	320.0［M+H］^+^	276.1， 302.1^*^	130	17， 25	NOR-D5
Fleroxacin（FLE）	C_17_H_18_F_3_N_3_O_3_	4.133	370.1［M+H］^+^	268.9， 326.0^*^	130	24， 20	FLE-D3
Ofloxacin（OFL）	C_18_H_20_FN_3_O_4_	4.135	362.0［M+H］^+^	261.0， 318.1^*^	140	29， 21	OFL-D3
Sulfamethazine（SMZ）	C_12_H_14_N_4_O_2_S	4.184	278.9［M+H］^+^	92.0， 185.9^*^	120	33， 17	/
Pefloxacin（PEF）	C_17_H_20_FN_3_O_3_	4.218	334.2［M+H］^+^	233.1， 290.1^*^	130	28， 16	PEF-D5
Ciprofloxacin（CIP）	C_17_H_18_FN_3_O_3_	4.238	332.1［M+H］^+^	314.1， 231.0^*^	130	21， 49	CIP-D8
Tetracycline（TET）	C_22_H_24_N_2_O_8_	4.385	445.2［M+H］^+^	154.0， 410.1^*^	120	22， 20	/
Lomefloxacin（LMX）	C_17_H_19_F_2_N_3_O_3_	4.391	352.1［M+H］^+^	308.1， 265.1^*^	130	18， 25	LMX-D5
Enrofloxacin（ENR）	C_19_H_22_FN_3_O_3_	4.584	360.1［M+H］^+^	342.1， 316.1^*^	130	25， 21	ENR-D5
Difloxacin（DFH）	C_21_H_19_F_2_N_3_O_3_·HCl	5.136	400.1［M+H］^+^	299.1， 382.1^*^	135	30， 20	DFH-D3
Sulfamethoxazole（SMX）	C_10_H_11_N_3_O_3_S	5.462	254.0［M+H］^+^	155.9， 92.0^*^	104	17， 29	/
Florfenicol（FF）	C_12_H_14_Cl_2_FNO_4_S	5.489	375.0［M+NH_4_］^+^	340.0， 241.0^*^	105	14， 25	/
Chlortetracycline（CTC）	C_22_H_23_ClN_2_O_8_	5.582	479.1［M+H］^+^	462.0， 444.0^*^	135	16， 22	/
Erythromycin（ERY）	C_37_H_67_NO_13_	6.522	734.4［M+H］^+^	576.3， 158.0^*^	155	17， 29	/
Dehydrated erythromycin（ERY-H_2_O）	C_37_H_65_NO_12_	6.876	716.4［M+H］^+^	558.4， 158.1^*^	150	25， 9	/
Clarithromycin（CTM）	C_38_H_69_NO_13_	7.084	748.4［M+H］^+^	590.3， 158.0^*^	165	21， 29	/
Roxithromycin（ROX）	C_41_H_76_N_2_O_15_	7.153	837.4［M+H］^+^	558.3， 679.3^*^	170	25， 21	/
NOR-D5	C_16_H_13_D_5_FN_3_O_3_	4.123	325.1［M+H］^+^	307.1^#^	130	25	
FLE-D3	C_17_H_15_D_3_F_3_N_3_O_3_	4.133	373.1［M+H］^+^	269.1， 329.2^*^	130	30， 20	
OFL-D3	C_18_H_17_D_3_FN_3_O_4_	4.134	365.1［M+H］^+^	261.1， 321.1^*^	130	30， 21	
CIP-D8	C_17_H_10_D_8_FN_3_O_3_	4.197	340.1［M+H］^+^	296.1， 322.1^*^	130	20， 30	
PEF-D5	C_17_H_15_D_5_FN_3_O_3_	4.198	339.1［M+H］^+^	295.1， 321.1^*^	130	19， 22	
LMX-D5	C_17_H_14_D_5_F_2_N_3_O_3_	4.371	357.1［M+H］^+^	313.1， 270.1^*^	130	18， 25	
ENR-D5	C_19_H_17_D_5_FN_3_O_3_	4.582	365.1［M+H］^+^	321.1^#^	130	21	
DFH-D3	C_21_H_16_D_3_F_2_N_3_O_3_·HCl	5.135	403.1［M+H］^+^	299.1， 385.2^*^	135	33， 23	

CEs： collision energies； *： quantitative ion； #： both qualitative and quantitative ions.

### 1.5 质量控制与保证

实验所用器皿均依次用自来水和超纯水洗净，并烘干备用。在样品处理过程中，设置过程空白实验，以监测实验过程中是否存在污染问题。在样品分析过程中，通过测定溶剂空白样和质控样，对背景污染情况以及仪器状态进行监测。

## 2 结果与讨论

### 2.1 样品提取条件的优化

#### 2.1.1 提取溶剂的选择

22种目标抗生素呈弱极性或中等极性，基于此，选择弱极性的有机溶剂（甲醇或乙腈）来进行提取；同时，利用缓冲溶液调整抗生素的等电状态，以提高抗生素的溶解度^［25］^。本研究以空白加标（5 ng/g）的沉积物样品为研究对象，按1.3节方法进行前处理，以目标化合物的峰面积为指标，分别考察了甲醇-磷酸盐缓冲溶液（1∶1，v/v）、甲醇-EDTA/McIlvaine缓冲溶液（1∶1，v/v）、甲醇-柠檬酸缓冲溶液（1∶1，v/v）、乙腈-磷酸盐缓冲溶液（1∶1，v/v）、乙腈-EDTA/McIlvaine缓冲溶液（1∶1，v/v）、乙腈-柠檬酸缓冲溶液（1∶1，v/v）、50%乙腈甲醇-磷酸盐缓冲溶液（1∶1，v/v）、50%乙腈甲醇-EDTA/McIlvaine缓冲溶液（1∶1，v/v）、50%乙腈甲醇-柠檬酸缓冲溶液（1∶1，v/v）9种提取溶剂对22种目标化合物的提取效果。实验结果表明，对于FF，9种提取溶剂的提取效果相差不大；对于四环素类抗生素，50%乙腈甲醇-EDTA/McIlvaine缓冲溶液和乙腈-EDTA/McIlvaine缓冲溶液均有较好的提取效果，但乙腈-EDTA/McIlvaine缓冲溶液的提取效果更佳；对于CTM、ERY-H_2_O、ROX这几种大环内酯类抗生素，50%乙腈甲醇-磷酸盐缓冲溶液、50%乙腈甲醇-EDTA/McIlvaine缓冲溶液、乙腈-磷酸盐缓冲溶液和乙腈-EDTA/McIlvaine缓冲溶液均有较好的提取效果；对于喹诺酮类抗生素，乙腈-磷酸盐缓冲溶液和乙腈-EDTA/McIlvaine缓冲溶液的提取效果较好，但乙腈-EDTA/McIlvaine缓冲溶液的提取效果更佳；对于大部分磺胺类抗生素，乙腈-EDTA/McIlvaine缓冲溶液的提取效果最好。综合考虑，实验最终选择乙腈-EDTA/McIlvaine缓冲溶液（1∶1，v/v）作为提取溶剂。吕凯等^［26］^建立了一种用于测定水和沉积物中14种抗生素的检测方法，在前处理环节中，该方法同样采用乙腈-EDTA/McIlvaine缓冲溶液（1∶1，v/v）作为沉积物中抗生素的提取溶剂，与本研究的优化结果一致。

#### 2.1.2 提取次数的优化

在其他提取条件固定的情况下，考察了不同提取次数对22种目标化合物提取效果的影响。与提取1次相比，当提取次数为2次时，大多数目标化合物的峰面积没有明显提升；当提取次数为3次时，大部分目标化合物的峰面积都有提升。具体而言，对于5种喹诺酮类抗生素（CIP、DFH、FLE、LMX和NOR），提取3次时的峰面积比提取1、2次时分别增加了9.3%~17.1%和9.4%~20.0%；对于4种大环内酯类抗生素（CTM、ERY、ERY-H_2_O和ROX），提取3次时的峰面积比提取1、2次时分别增加了1.7%~19.3%和9.9%~14.3%；对于3种四环素类抗生素（CTC、OTC和TET），提取3次时的峰面积比提取1、2次时分别增加了22.5%~40.8%和18.0%~27.5%；对于5种磺胺类抗生素（SDZ、SMR、SMX、SMZ和TMP），提取3次时的峰面积比提取1、2次时分别增加了9.6%~23.0%和7.1%~21.9%。此外，对于2种喹诺酮类抗生素（ENR和OFL）和1种氯霉素类抗生素（FF），提取次数对提取效果影响不大；对于1种喹诺酮类抗生素（PEF）和1种磺胺类抗生素（STZ），提取2次时效果最好。对于四环素类抗生素，其极性较强且结构中含有多个可电离基团，更容易被沉积物吸附，因此提取3次时峰面积增加的最多。综上，本研究选择提取次数为3次。

### 2.2 在线SPE条件的优化

#### 2.2.1 在线SPE柱的选择

由于沉积物样品的基质较为复杂，选择合适的SPE柱对于降低基质效应、有效富集和净化目标化合物至关重要。目前，用于富集和净化环境样品中抗生素的SPE柱类型主要有PLRP-S柱^［27］^、HLB柱^［28］^和C18柱等。本研究以22种抗生素混合标准溶液（1 µg/L）的峰面积大小为指标，考察了4种在线SPE柱（PLRP-S柱（12.5 mm×2.1 mm，15 µm）、PLRP-S柱（12.5 mm×4.6 mm，15 µm）、HLB柱（20 mm×3.0 mm，5 µm）和5TC-C18柱（12.5 mm×4.6 mm，5 µm））对22种目标化合物的在线富集效率。实验结果表明，4种SPE柱均能不同程度地富集抗生素，但5TC-C18柱对半数以上抗生素的富集效率均低于其他3种SPE柱；PLRP-S柱（12.5 mm×4.6 mm，15 µm）对5种磺胺类抗生素（SDZ、SMR、SMX、SMZ和STZ）和3种四环素类抗生素（CTC、OTC、TET）的富集效率明显低于PLRP-S柱（12.5 mm×2.1 mm，15 µm）和HLB柱；PLRP-S柱（12.5 mm×2.1 mm，15 µm）和HLB柱对大部分抗生素的富集效率相差不大，但PLRP-S柱（12.5 mm×2.1 mm，15 µm）对PEF、SMR、SMZ和TET的富集效率明显高于HLB柱。整体来看，PLRP-S柱（12.5 mm×2.1 mm，15 µm）对大部分抗生素的富集效率更高一些。Axel等^［27］^同样采用PLRP-S柱（12.5 mm×2.1 mm，15 µm）作为在线SPE柱，开发了一种在线SPE-LC-MS/MS法用于分析水环境中的17种抗生素。综上所述，本研究选择PLRP-S柱（12.5 mm×2.1 mm，15 µm）作为在线SPE柱，用于22种抗生素的在线富集与净化。

#### 2.2.2 初始样品加载流动相的优化

当样品溶液通过四元泵从进样器加载到在线SPE柱时，为保证在线SPE柱对目标化合物实现有效富集，四元泵的初始（0~2 min）加载流动相需具备非常弱的洗脱能力。因此，有必要对四元泵的初始加载流动相组成进行优化。本研究以22种抗生素混合标准溶液（1 µg/L）的色谱峰面积为考察指标，研究了3种不同比例（98∶2、96.5∶3.5、95∶5）的初始加载流动相（A_1_：0.1%甲酸水溶液（含2.5 mmol/L乙酸铵溶液），B_1_：甲醇）对目标化合物在SPE柱中富集效果的影响。结果表明，在3种不同初始加载流动相比例下，22种抗生素均能较好地保留在SPE柱上；但研究发现，当初始加载流动相体积比为95∶5时，SDZ和STZ的色谱峰开始变形展宽；在另两种初始流动相比例（98∶2和96.5∶3.5）下，22种抗生素的色谱峰面积相差不大，但当初始流动相体积比为98∶2时，半数以上抗生素的色谱峰面积略高一些，且峰形更加尖锐、对称。因此，本研究将四元泵的初始加载流动相比例设定为98∶2。在最佳实验条件下，22种目标抗生素的MRM色谱图见图3。

**图3:**
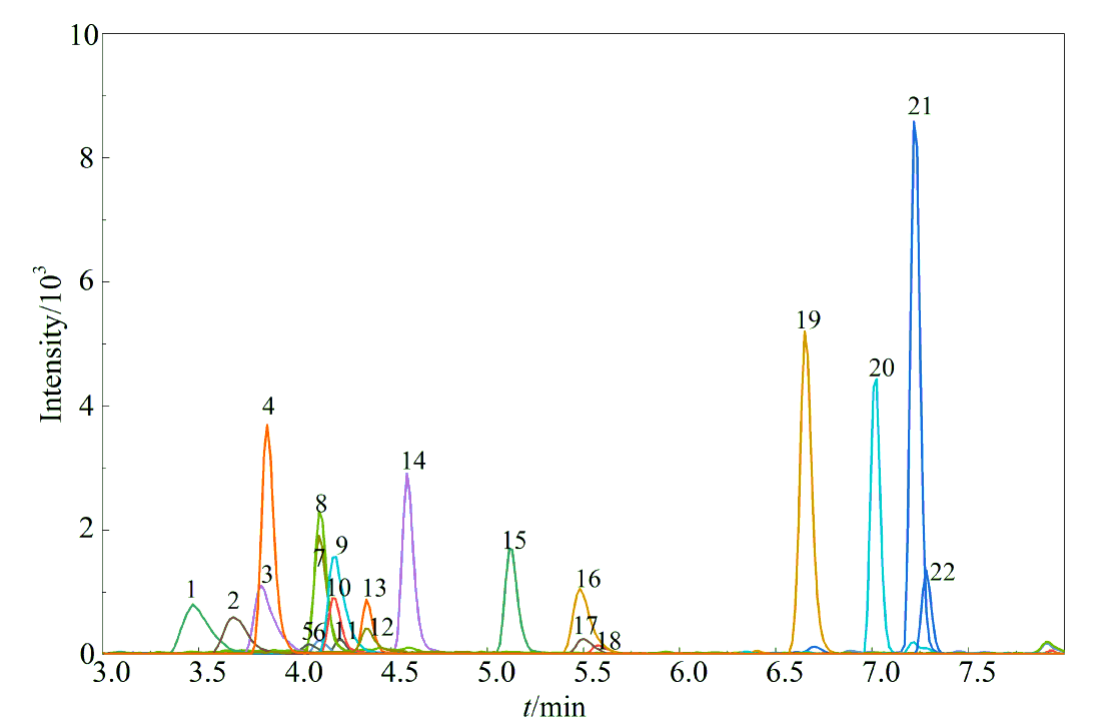
22种抗生素混合标准溶液（1 μg/L）的MRM色谱图 Peak identifications： 1. SDZ； 2. STZ； 3. SMR； 4. TMP； 5. OTC； 6. NOR； 7. FLE； 8. OFL； 9. SMZ； 10. PEF； 11. CIP； 12. TET； 13. LMX； 14. ENR； 15. DFH； 16. SMX； 17. FF； 18. CTC； 19. ERY； 20. ERY-H_2_O； 21. CTM； 22. ROX.

### 2.3 方法学考察

#### 2.3.1 基质效应

以空白沉积物样品为基质，按1.3.2节方法进行前处理，用空白基质提取液配制系列质量浓度的基质匹配混合标准溶液，同时配制相同质量浓度的溶剂混合标准溶液。根据文献［^29^］方法来评价基质效应（ME），即ME=（*A*_s_‒*A*_x_）/*A*_s_×100%。其中，*A*_x_为基质匹配混合标准溶液的峰面积，*A*_s_为溶剂混合标准溶液的峰面积。当|ME|<20%时，基质效应可忽略不计；当20%≤|ME|≤50%时，表示存在中等程度的基质效应；当|ME|>50%时，表示存在较强的基质效应。结果显示，22种目标化合物均存在不同程度的基质效应。其中，ERY、ERY-H_2_O、FF、OTC、SDZ、SMR、SMX、SMZ、STZ、TET这10种化合物的基质效应较强，其余目标化合物的基质效应均为中等程度或更低。因此，本实验采用基质匹配混合标准溶液对实际沉积物样品中的抗生素进行定量测定。

#### 2.3.2 线性范围、检出限与定量限

用空白沉积物样品的基质提取液配制系列质量浓度（0.5、1、2、4、5、7.5、10、25 ng/L）的基质匹配混合标准溶液（内标质量浓度均为5 ng/L），其中CTC、OTC和TET的质量浓度为其余抗生素的10倍。从低质量浓度到高质量浓度依次进样，上机检测。喹诺酮类抗生素采用内标法定量，其余抗生素采用外标法定量。对于8种喹诺酮类抗生素，以目标化合物的质量浓度为横坐标（*X*，ng/L），目标化合物与对应内标的定量离子峰面积之比为纵坐标（*Y*），绘制相应的标准曲线；对于其他14种抗生素，以目标化合物的质量浓度为横坐标（*x*，ng/L），目标化合物的定量离子峰面积为纵坐标（*y*），绘制相应的标准曲线。结果如表3所示，22种目标化合物在各自的质量浓度范围内线性关系良好，相关系数（*R* ^2^）均≥0.990 0。分别以3倍和10倍信噪比（*S*/*N*）计算LOD和定量限（LOQ），22种目标化合物的LOD为0.001~0.08 ng/g，LOQ为0.004~0.4 ng/g。

**表3:** 22种目标化合物的线性范围、线性方程、相关系数、检出限和定量限

Compound	Linear range/（ng/L）	Linear equation	*R* ^2^	LOD/（ng/g）	LOQ/（ng/g）
SDZ	0.5‒25	*y*=459.71*x*+0.33	0.9970	0.04	0.08
STZ	0.5‒25	*y*=592.93*x*+18.43	0.9946	0.02	0.04
SMR	0.5‒25	*y*=586.29*x*‒22.08	0.9934	0.04	0.08
TMP	0.5‒25	*y*=1051.10*x*‒29.21	0.9986	0.04	0.4
OTC	5‒250	*y*=695.39*x*‒450.82	0.9937	0.04	0.16
NOR	4‒25	*Y*=165.16*X*‒1.27	0.9927	0.004	0.04
FLE	2‒25	*Y*=615.94*X*‒99.01	0.9944	0.004	0.08
OFL	1‒25	*Y*=754.94*X*+146.03	0.9945	0.02	0.08
SMZ	0.5‒25	*y*=201.04*x*‒17.27	0.9940	0.04	0.08
PEF	2‒25	*Y*=126.27*X*‒9.86	0.9900	0.02	0.08
CIP	2‒25	*Y*=105.57*X*+1.52	0.9947	0.04	0.08
TET	5‒250	*y*=126.41*x*+11.38	0.9941	0.08	0.16
LMX	0.5‒25	*Y*=205.93*X*‒43.79	0.9910	0.004	0.04
ENR	1‒25	*Y*=666.73*X*+98.76	0.9922	0.02	0.04
DFH	0.5‒25	*Y*=375.66*X*+30.04	0.9990	0.004	0.04
SMX	0.5‒25	*y*=439.94*x*+7.33	0.9992	0.004	0.08
FF	0.5‒25	*y*=98.61*x*+16.18	0.9964	0.002	0.004
CTC	40‒250	*y*=49.53*x*‒16.87	0.9911	0.04	0.4
ERY	0.5‒25	*y*=392.43*x*+18.37	0.9992	0.002	0.004
ERY- H_2_O	0.5‒25	*y*=469.58*x*+15.03	0.9973	0.001	0.004
CTM	0.5‒25	*y*=578.81*x*+11.37	0.9972	0.001	0.004
ROX	0.5‒25	*y*=69.51*x*+2.66	0.9939	0.002	0.004

*Y*： peak area ratio of the target compound to the corresponding internal standard； *y*： peak area of the target compound； *X* and *x*： mass concentration， ng/L.

#### 2.3.3 回收率与精密度

为了考察所建方法的准确度，进行低、中、高3个水平的加标回收试验。向空白沉积物样品中添加低、中、高3个水平（1、5、25 ng/g）的22种抗生素混合标准溶液，其中CTC、OTC和TET的加标水平为其余抗生素的10倍。按1.3节方法进行前处理，每个样品平行处理3份，计算加标回收率和相对标准偏差（RSD）。结果如表4所示，在1 ng/g加标水平下，22种抗生素的回收率为45.1%~142.1%，其中5种喹诺酮类抗生素（CIP、FLE、LMX、NOR和OFL）的回收率较低（均<60%），可能是因为沉积物对喹诺酮类抗生素的吸附性较强，且加标水平过低导致这几种抗生素提取不完全。在5 ng/g加标水平下，22种抗生素的回收率为82.8%~145.6%；在25 ng/g加标水平，22种抗生素的回收率为83.5%~138.4%。本方法能够有效富集22种抗生素且精密度良好（RSD<14%），适用于实际海洋沉积物中22种抗生素的检测。

**表4:** 空白沉积物样品中22种抗生素在低、中、高加标水平下的回收率和精密度（*n*=3）

Compound	1 ng/g			5 ng/g			25 ng/g	
	Recovery/%	RSD/%		Recovery/%	RSD/%		Recovery/%	RSD/%
SDZ	139.1	9.5		143.3	3.4		132.2	2.0
STZ	61.4	7.7		128.5	2.9		123.1	1.5
SMR	132.3	5.0		139.0	3.8		127.7	0.8
TMP	94.1	7.7		100.5	2.7		102.7	1.5
OTC	117.0	7.0		121.5	6.0		103.8	5.1
NOR	45.2	2.9		98.6	4.6		103.2	3.1
FLE	49.8	5.7		99.6	4.8		102.4	3.2
OFL	57.7	13.6		99.2	6.5		110.0	6.6
SMZ	131.3	6.0		138.5	2.6		133.4	2.3
PEF	91.1	10.6		97.1	2.6		128.2	0.7
CIP	45.1	12.2		89.2	6.2		96.2	3.7
TET	107.7	3.0		107.0	5.7		99.1	3.3
LMX	52.3	7.9		99.0	4.8		102.8	1.4
ENR	62.0	6.3		98.1	4.2		102.3	3.0
DFH	65.2	4.8		99.0	2.1		101.2	2.9
SMX	140.0	13.4		145.6	3.9		138.4	2.7
FF	123.3	13.7		139.7	4.0		97.2	1.4
CTC	67.6	5.9		82.8	12.2		83.5	11.9
ERY	119.4	12.5		117.9	3.0		105.1	1.4
ERY-H_2_O	142.1	8.6		145.6	4.6		137.5	2.2
CTM	138.0	12.6		138.2	3.7		125.7	3.0
ROX	122.8	12.7		140.5	5.1		125.7	2.2

### 2.4 方法比对

将所建方法与其他文献方法进行比较，结果见表5。文献中的离线SPE方法一般将沉积物样品粗提取液稀释至300~350 mL，以3~6 mL/min的流速过离线SPE柱；而本方法则省略了这一步骤，且无需氮吹和复溶操作，大大缩短了样品前处理时间，减少了试剂消耗与抗生素的过程损耗；此外，本方法的在线SPE柱可重复使用，降低了分析成本。除此之外，本方法的回收率与其他文献方法相当，但LOD和LOQ更低，说明本方法具有良好的灵敏度和精密度，能够满足海洋沉积物中22种抗生素的定量检测要求。

**表5:** 本方法与文献方法的比较

Sample	Pretreatment	Volume^a^/mL	LOD/（ng/g）	LOQ/（ng/g）	Recovery/%	Ref.
Sediment	UAE-offline SPE	350	0.01‒0.45	-	40‒127	［^16^］
Sediment	ASE-offline SPE	300	0.034‒0.396	-	67.8‒109.8	［^17^］
Sediment	UAE-offline SPE	300	0.0055‒0.716	-	56.4‒110	［^30^］
Sediment	MAE-offline SPE	-	0.1‒3.8	0.3‒9.0	>60	［^31^］
Sediment	UAE-online SPE	0.2	0.001‒0.08	0.004‒0.4	45.1‒145.6	this method

a： the injection volume of the SPE column； UAE： ultrasound assisted extraction； ASE： accelerated solvent extraction； MAE： microwave assisted extraction； -： not mentioned.

### 2.5 实际海洋沉积物样品测定

利用所建方法测定山东近海四十里湾夏季和冬季沉积物样品中的抗生素，按1.3节方法进行样品的采集与提取，按1.4节方法进行在线SPE净化富集，之后进样分析，测定结果详见表6。四十里湾夏季沉积物中共检出5类19种抗生素，检出含量范围为0.01~34.64 ng/g，平均含量为0.01~6.31 ng/g，总含量为0.12~56.83 ng/g；冬季沉积物中共检出5类20种抗生素，检出含量范围为0.004~19.11 ng/g，平均含量为0.01~2.62 ng/g，总含量为0.05~26.24 ng/g。对于大多数抗生素而言，夏季沉积物中抗生素的检出含量平均值高于冬季沉积物，这可能与夏季是海水养殖的黄金季节，抗生素使用量增加有关。OTC是四十里湾夏季和冬季沉积物样品中总含量最高的抗生素。参考以往针对其他区域沉积物开展的研究^［32‒34］^，OTC在四十里湾夏季沉积物中的平均含量高于中国黄河三角洲地区（平均含量为4.80 ng/g）^［32］^和渤海辽东湾地区（平均含量为137.101 ng/kg）^［33］^，但低于南海海陵湾地区（平均含量为8.11 ng/g）^［34］^。相比于中国辽东湾^［33］^、渤海莱州湾^［9］^、长江口及邻近沿海等地区^［35］^沉积物中抗生素的分布特征，四十里湾夏、冬两季沉积物中检出的抗生素种类较多，但其含量处于中等偏下水平。综上所述，在山东近海四十里湾的夏季和冬季沉积物样品中均检出多种抗生素，表明四十里湾沉积物中抗生素的潜在风险需进一步评估。

**表6:** 山东近海四十里湾夏季和冬季沉积物样品中22种抗生素的检出结果 (ng/g)

Compound	Summer sediments（*n*=9）				Winter sediments（*n*=10）		
	Content range	Average content	Total content		Content range	Average content	Total content
SDZ	ND‒0.27	0.05	0.41		ND‒0.09	0.03	0.29
STZ	0.02‒1.72	0.31	2.76		ND‒0.35	0.09	0.89
SMR	ND‒0.06	0.01	0.13		ND‒0.03	0.01	0.13
TMP	0.07‒2.00	0.32	2.88		0.06‒0.12	0.09	0.88
OTC	2.66‒34.64	6.31	56.83		ND‒4.34	2.62	26.24
NOR	0.56‒1.44	0.88	7.94		0.18‒1.08	0.59	5.86
FLE	ND	/	/		ND	/	/
OFL	0.24‒5.73	2.68	24.13		ND‒19.11	2.52	25.20
SMZ	ND‒1.06	0.14	1.24		ND‒1.67	0.28	2.83
PEF	ND	/	/		ND	/	/
CIP	0.89‒3.15	1.83	16.51		0.74‒2.59	1.80	18.04
TET	ND‒3.27	0.43	3.91		ND‒0.78	0.18	1.77
LMX	ND	/	/		ND‒0.13	0.02	0.20
ENR	ND‒0.45	0.20	1.77		ND‒0.40	0.15	1.46
DFH	ND‒0.08	0.04	0.32		ND‒0.04	0.01	0.07
SMX	ND‒<LOQ	0.01	0.12		ND‒<LOQ	0.01	0.05
FF	0.05‒3.33	0.63	5.67		<LOQ‒2.29	0.93	9.33
CTC	ND‒2.32	0.26	2.32		ND‒2.13	0.59	5.94
ERY	0.01‒0.44	0.07	0.63		0.004‒0.16	0.07	0.69
ERY-H_2_O	0.04‒0.70	0.13	1.20		0.06‒0.52	0.14	1.42
CTM	0.04‒0.28	0.09	0.84		0.04‒0.29	0.11	1.06
ROX	0.17‒0.49	0.28	2.53		0.16‒0.61	0.32	3.18

ND： not detected.

## 3 结论

本文建立了在线SPE净化-UHPLC-MS/MS直接测定海洋沉积物中22种抗生素的检测方法，并成功将该方法应用于山东近海四十里湾夏季和冬季沉积物中抗生素的测定。该方法具有较高的灵敏度和精密度，能够精准检测海洋沉积物中常见的抗生素，从而为近海沉积物中抗生素的日常监测工作提供有力的技术支撑。
